# Oxygen-independent, CDK4/CDK6-dependent degradation of hypoxia-inducible factor-1α takes cancers’ breath away

**DOI:** 10.18632/oncotarget.28166

**Published:** 2022-01-05

**Authors:** Len Neckers

**Affiliations:** ^1^Urologic Oncology Branch, Center for Cancer Research, National Cancer Institute, NIH, Bethesda, MD 20892, USA

**Keywords:** hypoxia, HIF-1α degradation, cyclin dependent kinases, Smurf2, Hsp90

***Commentary on:** Zhao and El-Deiry. Identification of Smurf2 as a HIF-1α degrading E3 ubiquitin ligase. Oncotarget. 2021; 12:1970–79. https://doi.org/10.18632/oncotarget.28081. [PubMed]*

In a recent paper in Oncotarget [1], Zhao and El-Deiry report that SMAD specific E3 ubiquitin protein ligase 2 (Smurf2) can mediate oxygen-independent proteasomal degradation of hypoxia-inducible transcription factor-1α (HIF-1α), a subunit of the heterodimeric transcription factor HIF-1. Stabilization/upregulation of HIF-1α is important in tumor cells because it drives a transcriptional program that inhibits apoptosis, enhances cell migration and promotes angiogenesis, while also impacting the host anti-tumor immune response [2, 3].

At the protein level, HIF-1α expression is oxygen-dependent. In normoxia, HIF-1α protein is degraded by the proteasome, after ubiquitination by the von Hippel Lindau (VHL) E3 ligase. HIF-1α prolyl hydroxylation, in the presence of oxygen, iron and 2-oxoglutarate, creates a binding site for VHL. Thus, in hypoxia HIF-1α protein is protected from VHL binding and subsequent degradation. The importance of Zhao and El-Deiry’s finding is that, unlike VHL-mediated ubiquitination, Smurf2-mediated ubiquitination of HIF-1α is oxygen-independent and thus can occur in both normoxic and hypoxic cells and tumors.

The apparent mechanism underlying Smurf2-mediated ubiquitination and proteasomal degradation of HIF-1α is also significant. El-Deiry and colleagues previously described VHL-independent stabilization of HIF-1α by the cyclin-dependent kinases CDK1 and CDK4 [4]. In the current study, Zhao and El-Deiry demonstrate that pharmacologic inhibition of CDK1 or CDK4/CDK6 results in increased association of Smurf2 with HIF-1α and leads to proteasome-dependent reduction in HIF-1α protein expression in both normoxic and hypoxic conditions, suggesting that the HIF-1α stabilizing effect of the CDKs is due to oxygen-independent reduction in Smurf2-HIF-1α association. They confirmed the key role played by Smurf2 using Smurf2 knockdown, over-expression and pharmacologic inhibition. Further, they demonstrated that Smurf2 was able to modulate HIF-1α expression in clear cell renal cell carcinoma (ccRCC) cells lacking functional VHL, and their analysis of TCGA data demonstrated a positive correlation of *SMURF2* overexpression and increased disease-free survival and overall survival in ccRCC.

The CDK4/CDK6 inhibitors palbociclib, ribociclib and abemaciclib are US Food and Drug Administration (FDA)-approved for treatment of hormone receptor-positive, HER2-negative advanced or metastatic breast cancer after disease progression following endocrine therapy, and these drugs are currently being examined for efficacy in additional solid tumor models, including ccRCC and other cancers with HIF-1α deregulation [5]. Mechanistically, CDK4/CDK6 inhibitors prevent phosphorylation of the retinoblastoma (Rb) protein [3], leading to suppression of transcription of E2F target genes required for the timely regulation of cell cycle progression. Resistance to CDK4/CDK6 inhibitors is generally associated with loss of Rb and Rb expression has been proposed as a discriminatory biomarker for selection of patients likely to respond to CDK4/CDK6 inhibition. However, although CDK4/CDK6 inhibitor impact on HIF-1α requires Smurf2, it is independent of Rb status [2]. Thus, expression of HIF-1α, or its transcriptional targets may identify novel, Rb-independent prognostic biomarkers of response to these agents.

HIF-1α is a client of the molecular chaperone Hsp90 [6]. The RING E3 ubiquitin ligase Cullin 5 promotes ubiquitination and degradation of HIF-1α upon Hsp90 inhibition [7]. Like Smurf2, the activity of this E3 ubiquitin ligase is oxygen- and VHL-independent. CDK4/CDK6 are also Hsp90 clients and both palbociclib and ribociclib dissociate CDK4/CDK6 from Hsp90, as do Hsp90 inhibitors [8]. In agreement with these reports, Zhao et al. have shown that combined treatment with CDK4/CDK6 inhibitors and Hsp90 inhibitors demonstrate Rb- and VHL-independent synergy *in vitro* and *in vivo* in several normoxic and hypoxic tumor models [2]. The combinatorial impact on HIF-1α expression can be explained by each pathway’s dependence on a unique E3 ubiquitin ligase. These preclinical findings have supported development of a Phase 1b clinical trial evaluating the CDK4/CDK6 inhibitor palbociclib and the Hsp90 inhibitor pimitespib in advanced breast cancer patients who have progressed on palbociclib alone and in patients with treatment-refractory solid tumors with Rb deficiency [2].

Both CDK4/CDK6 inhibitor-mediated and Hsp90 inhibitor-mediated ubiquitination may depend on recruitment of client-specific E3 ligases depending on cell context. Indeed, E3 ubiquitin ligases comprise one of the largest classes of HSP90 clients [9]. Human cells express more than 700 E3 ubiquitin ligases. The recent availability of a comprehensive CRISPR-Cas9 knockout library of all human E3 ubiquitin ligases [10] will no doubt facilitate investigation of the possible contextual recruitment of specific E3 ligases upon Hsp90 or CDK4/CDK6 inhibition, while also providing further mechanistic understanding of alternative, VHL-independent strategies for HIF-1α regulation.
